# Prescreening of tumor samples for tumor-centric transcriptome analyses of lung adenocarcinoma

**DOI:** 10.1186/s12885-022-10317-9

**Published:** 2022-11-17

**Authors:** Nayoung Kim, Dasom Jeong, Areum Jo, Hye Hyeon Eum, Hae-Ock Lee

**Affiliations:** 1 Department of Microbiology, College of Medicine, The Catholic University of Korea, 222 Banpo-daero, Seocho-gu, Seoul, 06591 Republic of Korea; 2 Department of Biomedicine and Health Sciences, Graduate School, The Catholic University of Korea, 222 Banpo-daero, Seocho-gu, Seoul, 06591 Korea

**Keywords:** Single-cell RNA sequencing, Tumor-centric analysis, Tumor cell portions, Prescreening strategy, Lung adenocarcinoma

## Abstract

**Background:**

Single-cell RNA sequencing (scRNA-seq) enables the systemic assessment of intratumoral heterogeneity within tumor cell populations and in diverse stromal cells of the tumor microenvironment. Gain of treatment resistance during tumor progression or drug treatment are important subjects of tumor-centric scRNA-seq analyses, which are hampered by scarce tumor cell portions. To guarantee the inclusion of tumor cells in the data analysis, we developed a prescreening strategy for lung adenocarcinoma.

**Methods:**

We obtained candidate genes that were differentially expressed between normal and tumor cells, excluding stromal cells, from the scRNA-seq data. Tumor cell-specific expression of the candidate genes was assessed via real-time reverse transcription-polymerase chain reaction (RT-PCR) using lung cancer cell lines, normal vs. lung cancer tissues, and lymph node biopsy samples with or without metastasis.

**Results:**

We found that CEA cell adhesion molecule 5 (*CEACAM5*) and high mobility group box 3 (*HMGB3*) were reliable markers for RT-PCR-based prescreening of tumor cells in lung adenocarcinoma.

**Conclusions:**

The prescreening strategy using *CEACAM5* and *HMGB3* expression facilitates tumor-centric scRNA-seq analyses of lung adenocarcinoma.

**Supplementary Information:**

The online version contains supplementary material available at 10.1186/s12885-022-10317-9.

## Background

Tumor heterogeneity is responsible for treatment resistance in cancer, involving outgrowth of pre-existing subclones or acquisition of resistance traits [1]. Single-cell genomic analysis provides a systemic tool for studying tumor heterogeneity at both DNA and RNA levels [2]. While DNA-level intratumoral heterogeneity can be addressed by variant allele frequencies in bulk sequencing data, RNA or gene expression level heterogeneity requires single-cell methods because of its quantitative nature. In early studies, large-scale single-cell RNA sequencing (scRNA-seq) analyses of cancer focused on the primary tumor landscape, depicting both tumor and microenvironmental cell populations [3, 4]. Current applications have shifted to comparative studies of different regions, conditions, and patients to gain clinical insights into treatment resistance and patient stratification [5, 6], which substantiated the need for appropriate sample selection.

Lung adenocarcinoma is the major cancer type that benefits from molecular targeted therapies, including tyrosine kinase inhibitors targeting the epidermal growth factor receptor (EGFR) mutations or ALK, EMAP-like 4, and neurotrophic receptor tyrosine kinase fusions [7]. Patients harboring these somatic alterations and responding to targeted therapy eventually develop treatment resistance, and it is critical to understand the underlying mechanisms to achieve long-term survival [8]. For example, secondary EGFR mutations (T790M or C797S) confer resistance to EGFR-targeted tyrosine kinase inhibitors [9, 10]. Activation of the salvage signaling pathway in MET, hepatocyte growth factor, AXL, Hh, and insulin-like growth factor 1 receptor also leads to resistance to EGFR-targeted therapies [11]. Study designs to compare before and after molecular targeted therapies or in responders and non-responders provide valuable opportunities to understand the mechanisms of treatment resistance. One hurdle in such study designs is the absence of tumor cells in the specimens, which results in the exclusion of precious data [12]. Ensuring the presence of tumor cells before single-cell experiments can save time and resources.

Several strategies that determine the presence or proportion of tumor cells may serve different purposes. First, histological evaluation of tissue sections is the standard diagnostic process for determining tumor type and stage [13]. Second, computational methods estimate tumor purity from genomic data at both the DNA and RNA levels. For example, the ABSOLUTE [14] algorithm infers tumor purity and ploidy from somatic DNA alterations in whole-genome sequencing data. Purity and ploidy information are critical for determining sub-clonal structures and tumor evolution. In comparison, the ESTIMATE [15] method uses gene expression data to infer tumor cellularity and stromal/immune cell fractions. Third, flow cytometry or real-time polymerase chain reaction (PCR) can be used to monitor micrometastases [16] or minimal/measurable residual disease during or after leukemia treatment [17]. The detection sensitivity of PCR-based methods is typically less than 0.01% [18], which is much higher than that of histological evaluation or genomic inference studies. The high sensitivity and simple experimental procedure that can be incorporated into the scRNA-seq pipeline make the real-time PCR approach the preferred prescreening method.

In this study, we aimed to develop a sample selection strategy for lung adenocarcinoma for tumor-centric analysis of scRNA-seq data. First, target gene selection was achieved using public scRNA-seq data, by cell type specification and differentially expressed gene analysis focusing on tumor cells. We then tested the candidate gene expression using real-time PCR in lung cancer cell lines, normal vs. tumor tissues, and lymph nodes with or without metastasis. Among the four candidate genes, CEA cell adhesion molecule 5 (*CEACAM5*) and high mobility group box 3 (*HMGB3*) distinguished the tumor from normal tissues and recapitulated tumor cellularity in single-cell transcriptome data. Based on these results, we recommend sample prescreening using multigene real-time PCR for beta-actin (*ACTB*)*, CEACAM5*, and *HMGB3* to ensure the presence of tumor cells.

## Methods

### Human specimens

The present study was reviewed and approved by the Institutional Review Board (IRB) of the Samsung Medical Center (SMC, Seoul, Korea) (IRB no. 2010–04–039-052). The individuals in this manuscript have given written informed consent. Tumor, distant normal lung, and normal lymph node tissues were obtained during conserving surgery at the SMC from seven patients diagnosed with lung cancer. Metastatic lymph nodes were collected from patients with lung cancer using endobronchial ultrasound and bronchoscopy. A total of 14 samples were collected and immediately snap-frozen in liquid nitrogen or dissociated.

### Human cancer cell lines

The human non-small cell lung cancer (NSCLC) cell lines A549 (CCL-185), NCI-H2228 (CRL-5935), HCC827 (KCLB70827), HCC1588 (KCLB71588), NCI-H854 (KCLB90854), HCC1833 (KCLB 71833) and HCC1195 (KCLB71195) were purchased from American Type Culture Collection (Manassas, VA, USA) and Korean Cell Line Bank (Seoul, Korea). Each cell line was cultured in the Roswell Park Memorial Institute-1640 medium (22400–089; Gibco, Waltham, MA, USA) supplemented with 10% fetal bovine serum (16000–044; Gibco, Waltham, MA, USA) at 37 °C in 5% CO2.

### RNA extraction and cDNA synthesis

Total RNA was extracted from the samples using the Qiagen RNeasy mini kit reagent (74104; Qiagen, Hilden, Germany), according to the manufacturer’s instructions. The quantity and quality of extracted RNA were assessed using a NanoDrop 2000 spectrophotometer (Thermo Scientific, Wilmington, DE, USA). cDNA was synthesized with an appropriate amount of RNA using the ReverTra AceTM qPCR RT Kit (TOFSQ-101; TOYOBO Co., Ltd., Osaka, Japan), according to the manufacturer’s recommendations. After RNA denaturation at 65 °C for 5 min, 1 μg of total RNA was diluted in 10 μL of reaction mixture containing 2 μL 5X RT buffer, 0.5 μL enzyme mix, 0.5 μL Primer mix, and water. The reaction mixture was incubated at 37 °C for 15 min. The cDNA product was further diluted four-fold with RNase-free water and used directly for real-time PCR.

The amplified cDNA samples were obtained in the library preparation step using Chromium Single Cell 5′ Library & Gel Bead Kit v1.1 (scRNA-Seq) [19] and Chromium Single Cell 3′ Library & Gel Bead Kit v3 (snRNA-Seq), according to the manufacturer’s recommendations.

### Real-time quantitative PCR

Real-time PCR was performed in a 96-well reaction plate (HSP9601; Bio-Rad Laboratories, Hercules, CA, USA) sealed with an adhesive film (MSB1001; Bio-Rad Laboratories, Hercules, CA, USA). Expression analysis of gene of interest (GOI) was performed using the Bio-Rad CFX96 Touch system and PrimeTime Gene Expression Master Mix (1055770; IDT, Coralville, IA, USA) with a predesigned primer and probe mix (Supplementary Table 1). Real-time PCR was performed according to the manufacturer’s instructions. All PCR were run in duplicate, and a non-template control was used for each run. Raw real-time PCR data were analyzed using CFX Manager 3.1, (1845000; Bio-Rad Laboratories, Hercules, CA, USA; https://www.bio-rad.com/ko-kr/sku/1845000-cfx-manager-software?ID=1845000) and PCR replication efficiency and CT numbers were obtained for each reaction. Raw data were transformed into a standard input format for plotting. Microsoft Excel was used to calculate the mean C_q_, ΔC_q_, ΔΔC_q,_ fold change, and log(fold change + 1): ΔC_q_ = C_q GOI_ – C_q *ACTB,*_ ΔΔC_q_ = ΔC_q GOI_ – Normal group ΔC_q_ value within the same batch. Relative fold change was determined using 2^-ΔΔCT^.

### Acquisition and analyses of single-cell and bulk RNA-seq data

Raw unique molecular identifier (UMI) gene-cell-barcode matrix derived from single-cell RNA sequencing data from patients with lung adenocarcinoma and their cell identity was downloaded from the National Center for Biotechnology Information Gene Expression Omnibus database (GSE131907) [19]. The UMI count for genes in each cell was log-normalized using the *NormalizeData* function of the *Seurat* R package [20].

RNA sequencing data for 1019 human cancer cell lines were obtained from the Cancer Cell Line Encyclopedia (CCLE) depmap portal (https://depmap.org/portal/download/) [21]. Expression levels were normalized as (log2 RPKM + 1), where RPKM represents reads per kilobase of transcript per million mapped reads for the genes in each sample.

RNA sequencing data from lung adenocarcinoma (LUAD) and lung squamous cell carcinoma (LUSC) samples were obtained from The Cancer Genome Atlas (TCGA) data portal (https://portal.gdc.cancer.gov/) [22]. This dataset included 533 primary tumor and 59 normal samples from TCGA LUAD and 502 primary tumor and 49 normal samples from TCGA LUSC. Expression levels were quantified as (log2 FPKM-UQ + 1), where FPKM-UQ refers to the upper quartile fragments per kilobase per million mapped reads for genes in each sample. Violin plots of gene expression for tumor and normal samples were generated using the *geom_violin* function of the *ggplot2* R package.

### Selection of tumor-specific genes

Significantly expressed genes for early-stage lung tumor (tLung), late-stage lung tumor (tL/B), and metastatic lymph node (mLN) compared to normal lung (nLung) were identified using the *FindMarkers* function (default parameters) of the *Seurat* package. Genes that were differentially expressed in each sample group were listed using the *FindAllMarkers* function (default parameters) in the *Seurat* package. The Wilcoxon rank-sum test with Bonferroni correction was used to calculate the significance of differences. We selected genes with log fold change > 0.25, *p*-value < 0.01, and adjusted *p*-value (Bonferroni) < 0.01, considering the fraction of expressing cells (> 25% of cells in either cell group, denoted as pct).

All methods were performed in accordance with the relevant guidelines and regulations.

## Results

### Schematic to identify genes for tumor prescreening

**Fig. 1:**
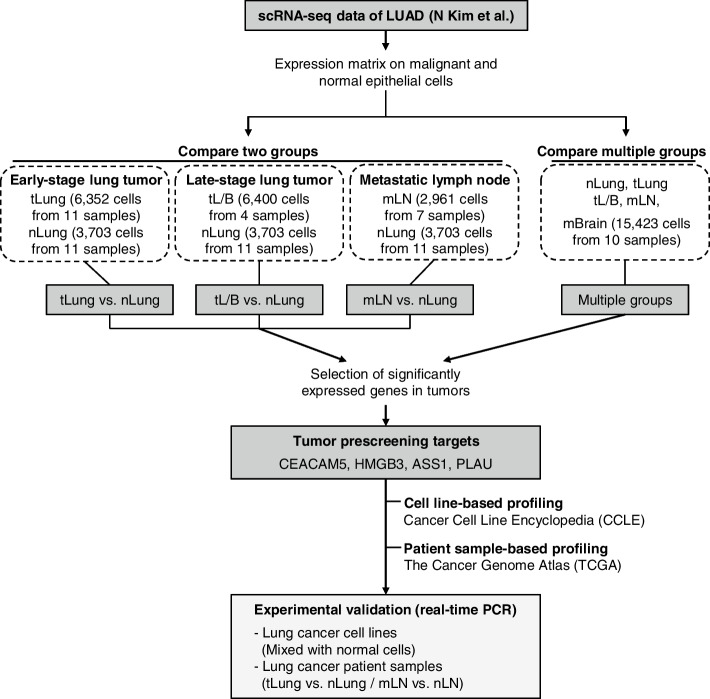
Tumor-centric single-cell analysis to identify candidate genes for tumor prescreening. Flow chart summarizing single-cell transcriptome analysis strategies to identify genes for tumor prescreening

Single-cell RNA sequencing data generated from normal or tumor tissues of patients with lung adenocarcinoma [19] were used to identify target genes indicative of tumor cell presence or proportions. For tumor-centric analysis, we extracted gene expression data only for malignant cells present in the tumor and compared them with normal epithelial cells (Fig. 1). Malignant cells are derived from various sources, including primary lung tumors (tLung and tL/B), metastatic lymph nodes (mLN), or brain metastases (mBrain). Normal epithelial cells were obtained from distant normal tissues of patients with tumors (nLung). We applied two analytical strategies to increase the specificity of the prescreening target genes to determine the extent of tumor cells. First, pairwise comparisons between tumor and normal sample groups (tLung vs. nLung, tL/B vs. nLung, and mLN vs. nLung) focused on genes upregulated in tumor cells compared with normal epithelial cells. Second, multi-set comparisons among all sample groups scanned genes specifically expressed in each tumor group. Among the genes with statistical significance in both comparisons, candidates were refined to test for the presence of tumor cells by real-time PCR. The expression profiles of candidate genes were also checked using RNA-seq data for cancer cell lines (CCLE) [21] and lung cancer patients (The Cancer Genome Atlas, TCGA) [22]. This approach provides genes exhibiting tumor cell-specific expression, allowing for the prescreening of samples harboring lung cancer cells.

### Tumor cell-specific gene selection in lung cancer

**Fig. 2:**
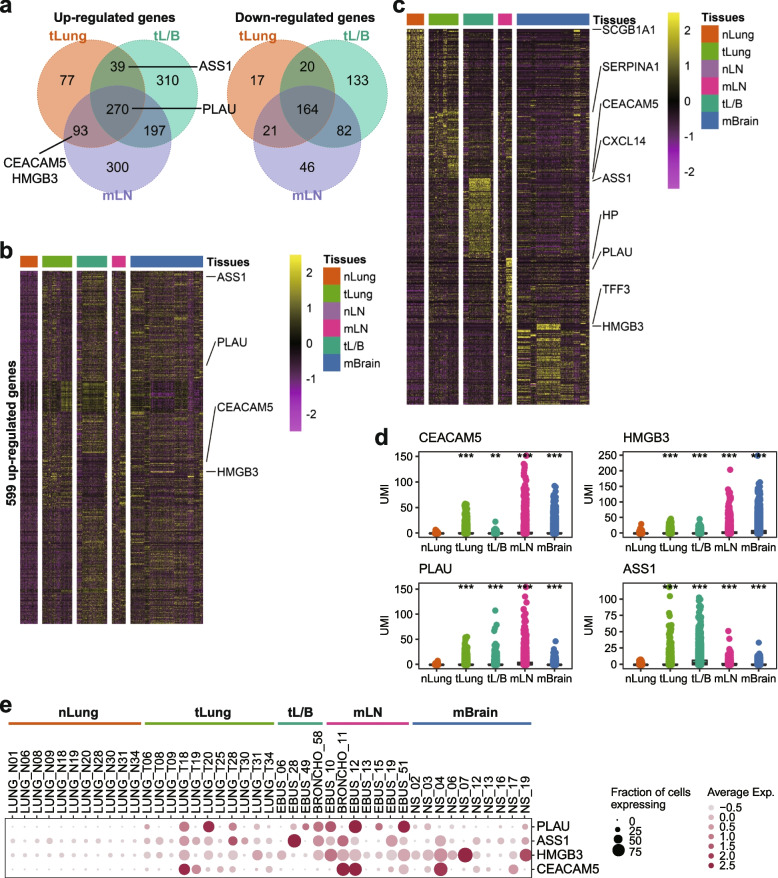
Identification of tumor-cell specific genes. **A** Venn diagram of up- and down-regulated genes for early-stage lung tumor (tLung), late-stage lung tumor (tL/B), and metastatic lymph node (mLN) compared to normal lung (nLung). **B** Expression map of 599 genes that were up-regulated in two or more tumor groups. Expression values scaled by z-transformation were used to a draw heatmap with limits of − 2.5 to 2.5. **C** Expression map of the top 100 genes upregulated for each sample group. Labels indicate the top-ranked and candidate genes. Expression values scaled by z-transformation were used to draw a heatmap with limits of − 2.5 to 2.5. **D** Expression plot of candidate genes for sample groups at the unique molecular identifier (UMI) level. Dot represents the UMI value for each single cell. Two-tailed Student’s *t*-test was performed. ***p-value < 0.001 and **p-value < 0.01. **E** Dot plot of candidate genes for each sample. Color indicates the average expression level. Size indicates the fraction of expressing cells

Following the schematics, we first listed the genes differentially expressed between malignant cells of the tumor (tLung, tL/B, and mLN) and normal epithelial cells (nLung) (Fig. 2A). Sets of 701, 1215, and 1173 genes were identified as significantly dysregulated in tumors (tLung, tL/B, and mLN, respectively) (Supplementary Table 2). Among them, 599 genes were significantly upregulated in tumor cells in at least two tumor groups compared to those in normal cells (Fig. 2B). Next, in the comparisons of multiple sample groups, we identified 3120 dysregulated genes specific to each sample group (Fig. 2C; Supplementary Table 2). We selected *CEACAM5, HMGB3*, plasminogen activator urokinase (*PLAU*), and argininosuccinate synthase 1 (*ASS1*) genes that were consistently denoted as the top-ranked upregulated genes in both comparisons. The association of lung cancer with selected tumor cell-specific genes, except *ASS1,* has been supported by previous studies. *CEACAM5* levels have been suggested to serve as prognostic determinants [23, 24] and have been correlated with metastatic lymph node tumor burden [16]. *HMGB3* expression was detected in circulating tumor cells in the peripheral blood of patients with lung cancer [25]. *PLAU* has been established as a prognostic marker for patients with lung cancer [26]. Tumor cell-specific expression of the selected genes was confirmed at the raw expression level (UMI) (Fig. 2D). These genes were overexpressed in tumor cells, with slight variations and low expression levels in all normal samples (Fig. 2E).

**Fig. 3:**
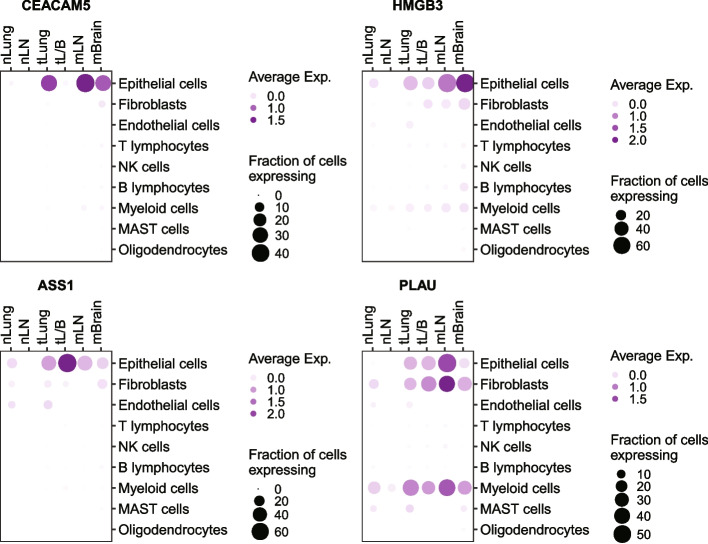
Expression of candidate genes in cell types. Dot plot of candidate genes for cell types in each sample group. Color indicates the average expression level. Size indicates the fraction of expressing cells

Target genes for the prescreening of tumor cells must have specific expression at cellular resolution. Prescreening using whole tumor tissue can be ambiguous if the gene is also expressed in the tumor stroma or in infiltrating immune cells. Therefore, the expression levels of candidate genes were compared between the cell types in each sample group (Fig. 3; Supplementary Fig. 1). The *CEACAM5, HMGB3*, and *ASS1* genes were specifically expressed in tumor cells from the tumor sample groups (tLung, tL/B, mLN, and mBrain). *PLAU* expression was detected not only in tumor cells, but also in fibroblasts and myeloid cells. These results indicate that *CEACAM5*, *HMGB3*, and *ASS1* are more reliable candidates than *PLAU* for the prescreening of tumor cells.

### Real-time PCR screening of lung cancer for tumor cell-specific gene expression

To confirm the expression of candidate genes in lung cancer specimens, we initially applied real-time RT-PCR (Supplementary Table 1) to the lung cancer cell lines A549, H2228, HCC827, HCC1195, HCC1588, and HCC1833 which were selected based on the CCLE (Supplementary Fig. 2A). Recapitulating the CCLE data, relatively high *PLAU* expression and low *CEACAM5* expression were detected in H2228 cells (Supplementary Fig. 2B). HCC827 and HCC1833 cells expressed high levels of *CEACAM5* (Supplementary Fig. 2C). To assess expression changes according to the tumor cell ratio, we spiked the cDNAs of H2228 cell line into those of normal lung tissues (Supplementary Fig. 2D). In the assessment of *HMGB3*, *PLAU*, and *ASS1*, the PCR products increased gradually with increasing amounts of H2228 cDNAs up to 60–80% and plateaued. Similarly, addition of HCC1833 cDNAs increased the *CEACAM5* signal (Supplementary Fig. 2E).

**Fig. 4:**
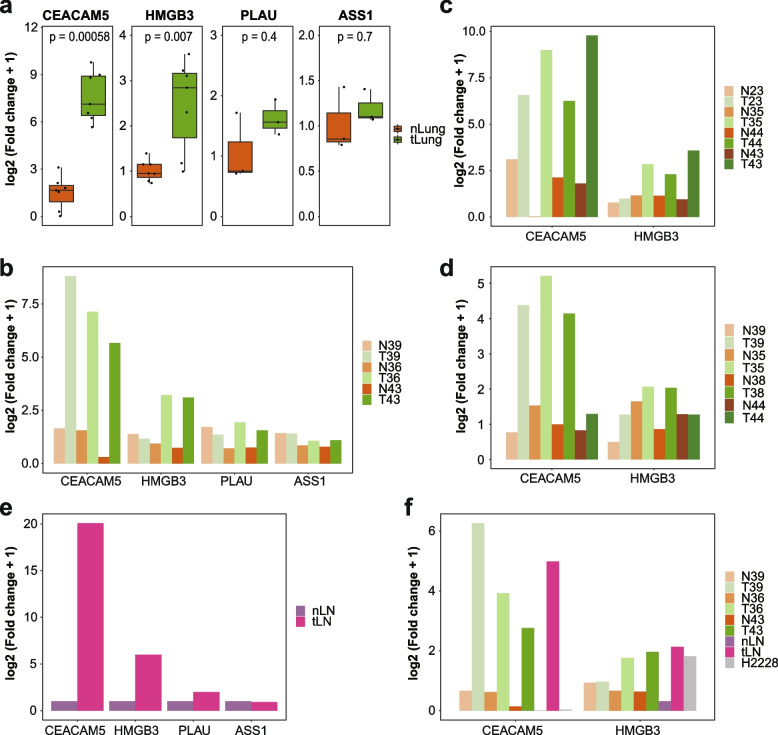
Real-time polymerase chain reaction (PCR) screening of prescreening candidates in lung tumor samples. **A** Box plot of candidate gene expression in tumor (T39, T36, T43, T23, T35, T44) and normal (N39, N36, N43, N23, N35, N44) lung samples. CEA cell adhesion molecule 5 (*CEACAM5*) and high mobility group box 3 (*HMGB3*) have two data points for samples T43 and N43, respectively. *P*-value was calculated by Wilcoxon rank sum test using *geom_signif* function of *ggplot2* package. **B** Expression levels of candidate genes in three tumor and three normal lung samples with individually synthesized cDNAs as template DNA. **C**, **D** Expression levels of *CEACAM5* and *HMGB3* in four tumor and four normal lung samples with cDNAs synthesized for **C** single-cell RNA sequencing (scRNA-seq) or **D** single-nucleus RNA sequencing (snRNA-seq). **E** Expression levels of candidate genes in metastatic (EBUS123) and normal (LN06) lymph node samples. **F** Gene expression levels of *CEACAM5* and *HMGB3* in human samples (four paired normal and tumor tissues) and cell line (H2228) were analyzed using the multiplex system

After the cell line test, we used non-small cell lung cancer (NSCLC) patient samples and compared target gene expression between the tumor and distant normal tissues (Fig. 4A). *CEACAM5* and *HMGB3* showed significant differences in expression between the two groups, and *PLAU* and *ASS1* showed slightly higher expression in tumor tissues, but the difference was not statistically significant. Differential expression between the tumor and normal samples was confirmed in various sample preparation stages and methods (Fig. 4B-D). Similarly, a difference in the expression levels of *CEACAM5* and *HMGB3* was observed in lymph node samples with or without metastasis (Fig. 4E). Pairwise comparisons of matched normal and tumor samples provided clearer decision criteria for tumor cell positivity. Without a matched normal sample, tumor positivity was determined for samples with > 10% tumor cell content (Supplementary Table 3). To apply the prescreening process as a single-tube reaction, we performed multiplex RT-PCR analyses using *CEACAM5*, *HMGB3*, and *ACTB* probes with different fluorescence dye formats, which resulted in consistent tumor-specific detection (Fig. 4F).

Altogether, these results suggest that real-time PCR screening of *CEACAM5* and *HMGB3* can be used to confirm the presence of tumor cells in lung adenocarcinoma specimens of both tissue and lymph node origin, as well as in cDNAs and single-cell or nuclear RNA sequencing libraries.

### Validation of tumor-specific gene expression using public datasets

**Fig. 5:**
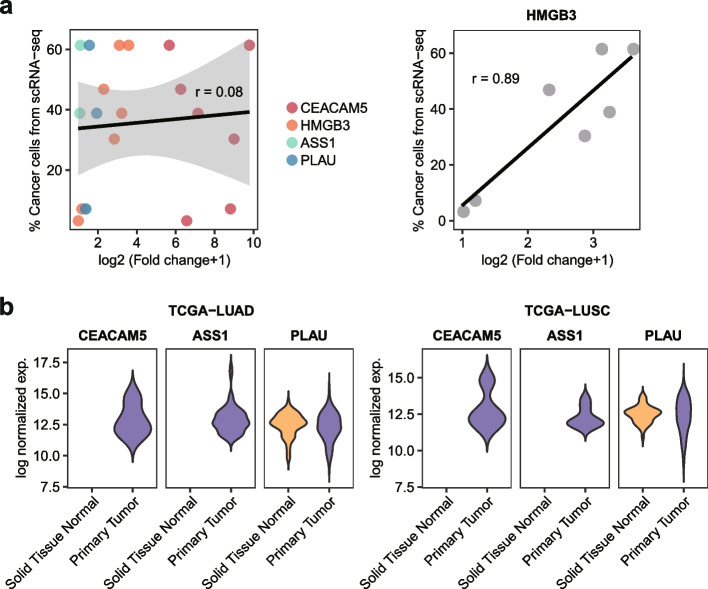
Tumor-specific expression of candidate genes in single-cell and bulk RNA-seq data. **A** Correlation of candidate genes (left) and *HMGB3* (right) between expression levels measured via real-time PCR and the percentage of tumor cells from scRNA-seq in tumor lung samples. *N* = 7 for *CEACAM5* and *HMGB3*, *N* = 3 for *ASS1* and *PLAU*. **B** Violin plots of *CEACAM5*, *ASS1*, and *PLAU* for tumor and normal samples from The Cancer Genome Atlas (TCGA) lung adenocarcinoma (LUAD) and lung squamous cell carcinoma (LUSC)

To further investigate whether the expression levels predicted the proportion of tumor cells, we calculated the correlation between gene expression levels measured by real-time PCR and the percentage of tumor cells obtained from single-cell sequencing data [19] (Fig. 5A). Overall, the four candidate genes showed a positive association, yet the correlation coefficient was small, likely because of the large variation in cellular expression levels. Among them, *HMGB3* expression showed the highest correlation with the tumor cell proportion.

Next, we examined the lung cancer cohort from TCGA [22] to determine differential expression of the four genes between normal and tumor at the bulk tissue level. As shown in Fig. 5B, *CEACAM5* and *ASS1* were specifically expressed in the lung tumor samples. *HMGB3* transcripts were not detected in any of the samples, and PLAU expression was not significantly different between the normal and tumor tissues. These data demonstrate the variation in sensitivity and specificity among the different gene detection and sample preparation methods. Taken together, the detection of *CEACAM5* and *HMGB3* by real-time PCR was suitable for sample prescreening before single-cell or nuclear sequencing experiments requiring the presence of tumor cells.

## Discussion

The power of single-cell RNA sequencing has made this technique a mainstream tool in cell biology to study normal development and differentiation processes, and to define cellular alterations in diseases. There is a need for versatile data generation for hypothesis testing and appropriate sample selection; however, proper guidelines are not available. During the experimental design process, we encountered a situation in which the tumor cell content was too low to perform a tumor-centric data analysis.

To study a tumor subpopulation using a single-cell genomics approach, choices can be made on whether to sort and enrich the target population or to perform all-inclusive analysis after ensuring tumor cell presence [27]. Both approaches have their own merits, the latter requiring no prior knowledge for sorting and allowing inference of cellular interactions between the tumor cells and the support cells in the tumor microenvironment [28]. Cellular composition in the tumor microenvironment and communication with tumor cells changes over time during tumor progression, metastasis, and treatment resistance. Therefore, the unsorted study design ensuring tumor cell presence in the microenvironmental context helps to elucidate disease-associated alterations of the tumor and support cell interactions, which could be a good target for therapeutic intervention.

As a prescreening strategy to ensure tumor cell inclusion in lung adenocarcinoma, we selected four genes showing tumor cell-specific gene expression from publicly available scRNA-seq data and adopted real-time PCR on cDNAs or RNA sequencing libraries of the study objects. The simplicity and reliability of real-time PCR make it the preferred prognostic gene expression testing platform for early-stage breast cancer [29]. During candidate gene expression testing for lung cancer, we found unexpected discrepancies between scRNA-seq and real-time PCR results. These discrepancies may be explained by the different dynamic ranges of each gene detection method [30], individual cell or population level measurements, and cell- vs. tissue-level gene expression analysis. Since the aim of this study was to develop a sample selection strategy for single-cell or nuclear RNA sequencing analysis, *CEACAM5* and *HMGB3*, which showed the best results in cell-level data, were selected as the final target genes. The use of this sample selection strategy will facilitate the efficient design of tumor-centric single-cell/nucleus genomic analyses.

## Conclusions

To guarantee tumor-centric analysis of lung cancer, we selected tumor cell-specific genes from the scRNA-seq data and performed real-time PCR to distinguish samples with or without tumor cell presence. We suggest *CEACAM5* and *HMGB3* as prescreening markers for single-cell or nuclear sequencing experiments.

## Supplementary Information

**Additional file 1: Supplementary Fig. 1.** t-distributed stochastic neighbor embedding (tSNE) plot colored based on the expression levels of candidate genes in each sample group.

**Additional file 2: Supplementary Fig. 2.** Detection sensitivity of prescreening candidates in the cancer cell population. (A) Waterfall plots of expression of candidate genes and beta-actin (*ACTB*) in Cancer Cell Line Encyclopedia (CCLE) cancer cell lines. (B) Bar plot of candidate genes in H2228 cells. (C) Relative expression of *CEACAM5* in six lung cancer cell lines compared to normal lung tissue. (D) Expression patterns of *HMGB3*, *PLAU*, and *ASS1* according to the mixing ratio of H2228 and normal lung tissue cDNAs. (E) Expression pattern of *CEACAM5* according to the mixing ratio of HCC1833 and normal lung tissue cDNAs.

**Additional file 3: Supplementary Table 1.** Predesigned primers for targeted polymerase chain reaction (PCR).

**Additional file 4: Supplementary Table 2.** List of differentially expressed genes specific to malignant lung cancer cells.

**Additional file 5: Supplementary Table 3.** Tumor cell percentage estimated via single-cell RNA sequencing (scRNA-seq) of patient samples.

## Data Availability

The datasets analyzed during the current study are available in the National Center for Biotechnology Information Gene Expression Omnibus database (GSE131907), CCLE depmap portal (https://depmap.org/portal/download/), and TCGA data portal (https://portal.gdc.cancer.gov/).

## Abbreviations

CEACAM5: CEA cell adhesion molecule 5
HMGB3: High mobility group box 3
PLAU: Plasminogen activator urokinase
ASS1: Argininosuccinate synthase 1
EGFR: Epidermal growth factor receptor
FBS: Fetal bovine serum
GOI: Gene of interest
RPKM: Reads per kilobase per million mapped reads
RT-PCR: Reverse transcription-polymerase chain reaction
scRNA-seq: Single-cell RNA sequencing
UMI: Unique molecular identifier
tLung: Early-stage lung tumor
tL/B: Late-stage lung tumor
nLung: Normal lung
mLN: Metastatic lymph node
mBrain: Metastatic brain
CCLE: Cancer Cell Line Encyclopedia
TCGA: The Cancer Genome Atlas
LUAD: Lung adenocarcinoma
LUSC: Lung squamous cell carcinoma
