# Supplementary figures and images for: Prescreening of tumor samples for tumor-centric transcriptome analyses of lung adenocarcinoma
Source: BMC Cancer. 2022 Nov 17;22:1186. doi: 10.1186/s12885-022-10317-9 (PMC9673386; doi:10.1186/s12885-022-10317-9)

nLung

tLung

mLN & tL/B

mBrain

nLN

CEACAM5

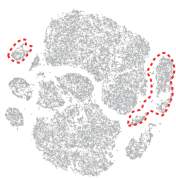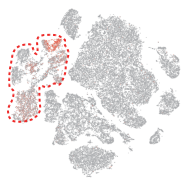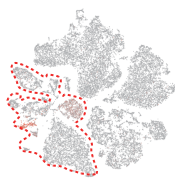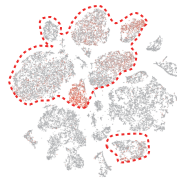

HMGB3

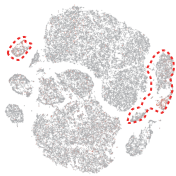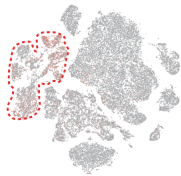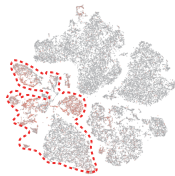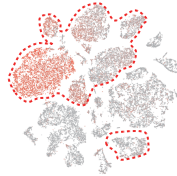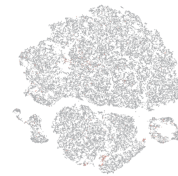

ASS1

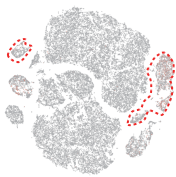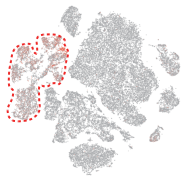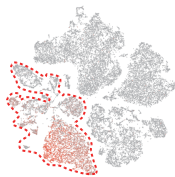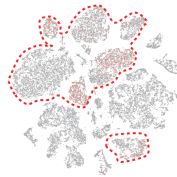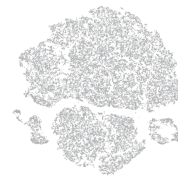

PLAU

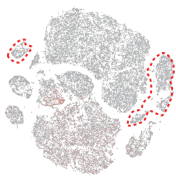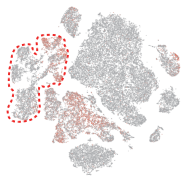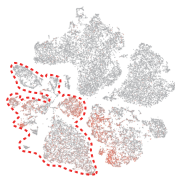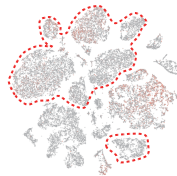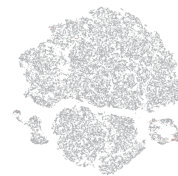

○ Epithelial cells

Supplement: Supplementary file 1 — Additional file 1: Supplementary Fig. 1. t-distributed stochastic neighbor embedding (tSNE) plot colored based on the expression levels of candidate genes in each sample group. [file 12885_2022_10317_MOESM1_ESM.pdf]
