# Supplementary material for: Prescreening of tumor samples for tumor-centric transcriptome analyses of lung adenocarcinoma
Source: BMC Cancer. 2022 Nov 17;22:1186. doi: 10.1186/s12885-022-10317-9 (PMC9673386; doi:10.1186/s12885-022-10317-9)
Supplement: Supplementary file 2 — Additional file 2: Supplementary Fig. 2. Detection sensitivity of prescreening candidates in the cancer cell population. (A) Waterfall plots of expression of candidate genes and beta-actin (ACTB) in Cancer Cell Line Encyclopedia (CCLE) cancer cell lines. (B) Bar plot of candidate genes in H2228 cells. (C) Relative expression of CEACAM5 in six lung cancer cell lines compared to normal lung tissue. (D) Expression patterns of HMGB3, PLAU, and ASS1 according to the mixing ratio of H2228 and normal lung tissue cDNAs. (E) Expression pattern of CEACAM5 according to the mixing ratio of HCC1833 and normal lung tissue cDNAs. [file 12885_2022_10317_MOESM2_ESM.pdf]

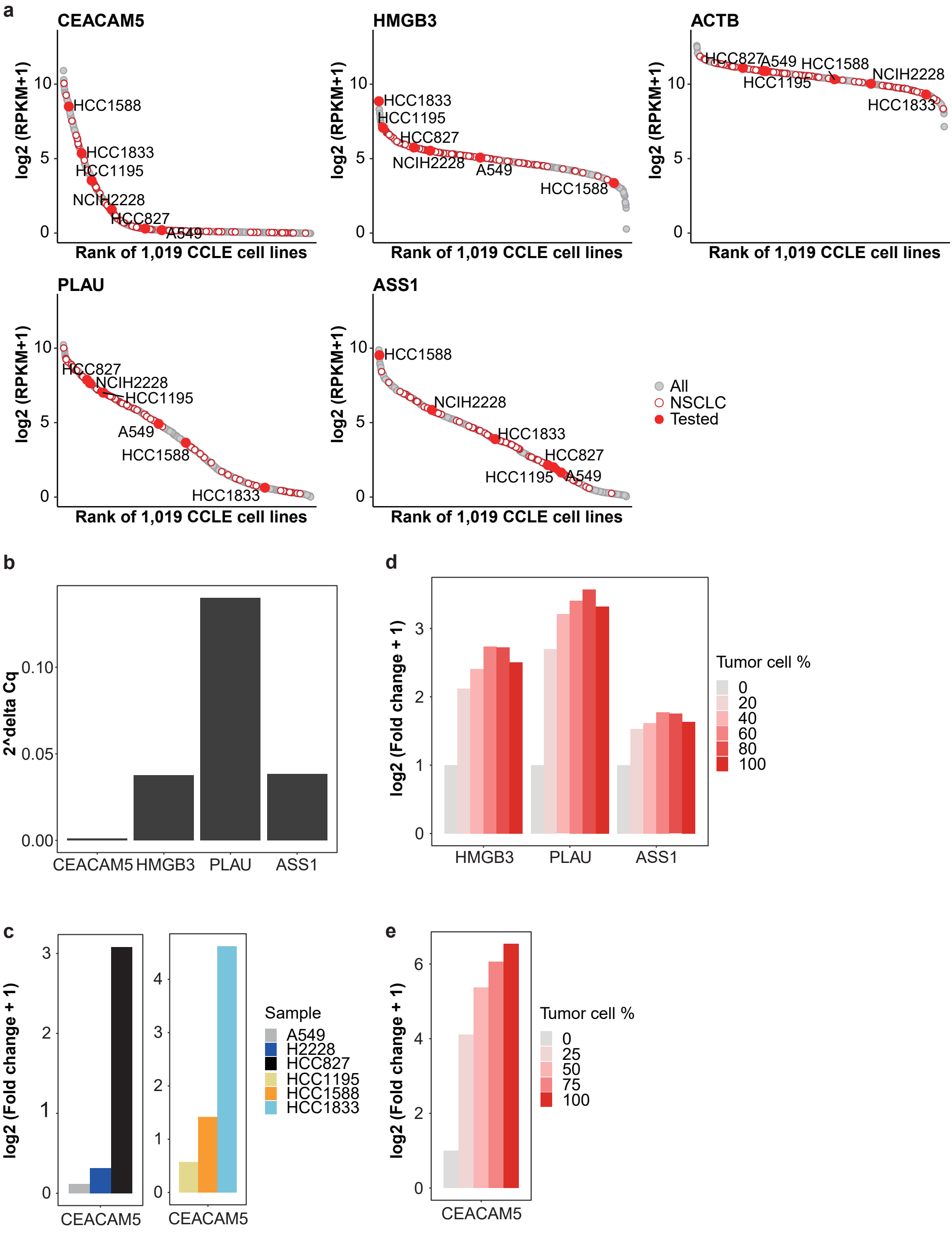
a
CEACAM5
HMGB3
ACTB
PLAU
ASS1
log2 (RPKM+1)
Rank of 1,019 CCLE cell lines
HCC1588
HCC1833
HCC1195
NCIH2228
HCC827
A549
All
NSCLC
Tested
b
2^delta Cq
0.00
0.05
0.10
CEACAM5 HMGB3 PLAU ASS1
c
log2 (Fold change + 1)
CEACAM5
Sample
A549
H2228
HCC827
HCC1195
HCC1588
HCC1833
d
log2 (Fold change + 1)
HMGB3
PLAU
ASS1
Tumor cell %
0
20
40
60
80
100
e
log2 (Fold change + 1)
CEACAM5
Tumor cell %
0
25
50
75
100
